# Disruptive effects of repeated stress on basolateral amygdala neurons and fear behavior across the estrous cycle in rats

**DOI:** 10.1038/s41598-019-48683-3

**Published:** 2019-08-23

**Authors:** Shannon R. Blume, Mallika Padival, Janice H. Urban, J. Amiel Rosenkranz

**Affiliations:** 1 Discipline of Cellular and Molecular Pharmacology, The Chicago Medical School, Rosalind Franklin University, North Chicago, IL 60064 USA; 2 Discipline of Physiology and Biophysics, The Chicago Medical School, Rosalind Franklin University, North Chicago, IL 60064 USA; 3 Center for Neurobiology of Stress Resilience and Psychiatric Disorders, The Chicago Medical School, Rosalind Franklin University, North Chicago, IL 60064 USA; 4 Present Address: AbbVie, Inc., 1 North Waukegan Road, North Chicago, IL 60064 USA

**Keywords:** Amygdala, Stress and resilience

## Abstract

Stress is a precipitating factor in depression and anxiety disorders. Patients with these disorders often show amygdala abnormalities. The basolateral amygdala (BLA) is integral in mood and emotion, and is sensitive to stress. While much is known about effects of stress on BLA neuron activity and morphology in males, less is known in females. We tested whether repeated stress exerts distinct effects on BLA *in vivo* neuronal activity and morphology of Golgi-stained BLA neurons [lateral (LAT) and basal (BA) nuclei] in adult female rats. Repeated restraint stress increased BLA neuronal firing and caused hypertrophy of BLA neurons in males, while it decreased LAT and BA neuronal firing and caused hypotrophy of neurons in the LAT of females. BLA neuronal activity and function, such as fear conditioning, shifts across the estrous cycle. Repeated stress disrupted this pattern of BLA activity and fear expression over the estrous cycle. The disruptive effects of stress on the pattern of BLA function across estrous may produce behavior that is non-optimal for a specific phase of the estrous cycle. The contrasting effects of stress may contribute to sex differences in the effects of stress on mood and psychiatric disorders.

## Introduction

Psychiatric disorders such as generalized anxiety disorder, major depressive disorder, and post-traumatic stress disorder (PTSD) afflict a huge number of people, the majority of which are female^1–6^. Patients suffering from these disorders often show functional abnormalities in limbic structures, particularly the prefrontal cortex, hippocampus and amygdala^7–10^. Stress not only can recapitulate these abnormalities observed in patients^11^, but can trigger the emergence of psychiatric disorders. However, the impact of stress on the development of psychiatric disorders may affect the sexes differently; for example, males typically experience more traumatic events, yet there is a higher incidence of PTSD in females^12^. Furthermore, limbic structures in men and women may be differentially sensitive to the effects of stress^1,3,6,12–15^.

Similarly in rodents, stress can exert contrasting effects on emotion-related behaviors of males and females^16^. Stress enhances fear conditioning performance in males^17–20^, whereas there are varied effects on fear learning in females, with reports of impaired^19^, no effect^21^, or enhanced fear learning in females^22,23^ that may vary depending on stressor, fear conditioning protocol or estrogen status.

The basolateral amygdala (BLA) plays a critical role in the expression and modulation of emotion, most notably measured as conditioned fear^24–28^. Differential effects of stress on fear conditioning may reflect different effects of stress on BLA function in females. Indeed, markers of cellular activation, such as cFos or other early genes and protein phosphorylation, demonstrate that acute stressors recruit the BLA^29–34^, and also hint at sex differences in the response of the BLA to stress^35–37^. More direct cellular measures have demonstrated that repeated stress increases firing of BLA neurons and produces hypertrophy of BLA dendrites in males^38–42^. However, very little is known about the cellular effects of repeated stress on BLA neurons in females. This first goal of this study was to test whether repeated restraint stress has different effects on BLA neuronal activity and morphology between male and female rats.

The BLA is comprised of several nuclei, including the lateral nucleus (LAT) and the basal nucleus (BA). These nuclei contribute to overlapping but different aspects of BLA-dependent behaviors, display differences in neuronal activity between males and females^43^ and may be differentially sensitive to stress^23,35–37^. Therefore, both of these nuclei will be separately examined in this study.

Mammalian biological rhythms, each with their own periodicities, influence a wide range of physiological processes in parallel with behaviors to produce adaptive benefits. Female mammals in the reproductive stages of life display ovarian cycles. This cycle consists of physiological changes in reproductive organs along with a coordinated modulation of sexual and affiliative behavior in a manner that optimizes reproductive success; a follicular phase (proestrus and estrus) in which behaviors are aimed at reproduction, and a luteal phase (metestrus and diestrus^44^). This includes several behaviors that rely on the amygdala, which can support or suppress sexual behaviors, such as anxiety and emotional memory^45–49^. Recent evidence demonstrates that BLA physiology and function shifts across the estrous cycle in rats such that LAT activity and LAT-dependent cued conditioned fear is greatest during diestrus while BA activity and BA-dependent contextual fear is greatest during proestrus^43^. The second goal of this study was to determine if repeated stress disrupts the pattern of BLA changes that usually occurs across the estrous cycle.

## Materials and Methods

All experimental procedures were carried out in accordance with the Guide for the Care and Use of Laboratory Animals^50^ and were approved by the Institutional Animal Care and Use Committee at Rosalind Franklin University of Medicine and Science. All appropriate efforts were made to minimize animal suffering, reduce the number of animals used, and when possible, to use alternatives to *in vivo* approaches.

### Animals and determination of estrous cyclicity

Male and female adult Sprague Dawley rats (average age on day of experimental measurement: female PND 77, male PND 76) (Charles River, Portage, MI) were group housed (3 rats/cage) with food and water available *ad libitum*. The animal room was maintained on a 12 hour reversed light-dark cycle at a limited range of temperature (68–79°F) and humidity (30–70%). Rats were allowed one week to habituate to the animal facility. Following 1 week of habituation, vaginal lavage was performed in female rats to monitor their estrous cycle. Cell cytology from lavage samples was examined microscopically as previously described^43,51^. Male rats were handled in a similar manner and for a similar amount of time to approximate handling of the females. Estrous cyclicity was monitored daily and followed a 4-day cycle pattern (2 days of diestrus, 1 day of proestrus, 1 day of estrus). Females with inconsistent estrous cycles were not included for these experiments. After 2 weeks of a consistent estrous cycle pattern (3 consecutive estrous cycles), females were initially selected based on estrous cycle phase such that they would be in either diestrus (low estrogen) or proestrus (high estrogen) in 10 days from this selection. This 10 day period bridges the time from initiation of control handling or repeated stress to the day at which experimental measures were taken (e.g. estrous females would be diestrus 1 in 10 days; diestrus 2 females would be proestrus in 10 days).

#### Assessment of uterine horns

After decapitation, visual inspection of the uterine horns was performed on all females as a secondary measure to confirm estrous cycle phase determined by vaginal lavage. During proestrus the uterus had a large, swollen, hyperemic appearance compared with those at diestrus^51^. To further validate estrous cycle phase, trunk blood was collected from a subset of females at the time of decapitation. Blood samples were immediately centrifuged, the serum collected and stored at −80 °C until quantification. Estradiol levels were quantified using an estradiol radioimmunoassay kit (sensitivity to 8 pg/mL; Coat-A-Count Estradiol Kit, Siemens Medical Solutions USA, Malvern, PA).

### Repeated restraint stress protocol

Male and female rats were randomly assigned to control or stress groups. Cohorts of control and stress rats were run in parallel whenever possible. Restraint stress was applied using a protocol that has been effective at inducing a change of BLA function^20,39^, as follows: Control handled animals were weighed and placed in a Plexiglas transfer cage with bedding for 20 minutes, on 7 out of 9 days. All control handling occurred in the animal housing room. Animals in the repeated stress group were weighed and placed in a transfer cage with no bedding and transported to a procedure room (80–90 lux overhead lighting). In the procedure room, animals were placed into a hemi-cylinder restraint tube for 20 minutes per day, for 7 out of 9 days. The hemi-cylinder restraint tubes were adjusted such that all animals had a similar degree of restricted trunk movement. One day following the final control or stress handling, animals were used for experimental measures. In a subset of rats, at autopsy, the adrenal gland was removed and wet weights were recorded.

### *In vivo* extracellular recording

Animals were anesthetized with urethane (1.5 g/kg i.p., Sigma Aldrich, St. Louis, MO) and placed in a stereotaxic device (David Kopf Instruments, Tujunga, CA). The level of anesthesia was monitored by periodic testing of the hind limb pedal reflex. Body temperature was monitored with a rectal temperature probe and maintained at ~37 °C with a heating pad (Model TC-1000, CWE Inc, Ardmore, PA). Prior to surgery, a local anesthetic was injected subcutaneous above the skull (0.3 mL, 1% lidocaine, Webster Veterinary Supply Inc., Devens, MA). A concentric bipolar stimulation electrode (David Kopf Instruments) was lowered into the medial prefrontal cortex (mPFC; +2.7 mm rostral (R), −0.7 mm L and −3.7 mm ventral (V) from bregma) to monitor the depth of anesthesia by recording ongoing rhythmic local field potentials. Single barrel electrodes (World Precision Instruments, Sarasota, FL) were made using a vertical microelectrode puller (PE-2; Narishige, Tokyo, Japan), and were broken under a microscope to a tip diameter of 1–2 μm and filled with a 2% Pontamine Sky Blue solution in 2M NaCl. The recording electrode was lowered into the BLA (−4.8 to −5.6 mm lateral (L), −2.8 to −3.8 mm caudal © from bregma and 6.5 to 9.0 mm V) using a hydraulic micromanipulator (model 640, David Kopf Instruments). Electrophysiological recordings started no earlier than 1 hour after the completion of surgery and electrode placements. The BLA includes the lateral nucleus (LAT) and the basal nucleus (BA). To ensure similar sampling of LAT and BA across animals, electrode penetrations through the BLA followed a predetermined grid of coordinates. During electrophysiological recordings a digital oscilloscope (2530 B&K Precision, Yorba Linda, CA) and audio monitor (AM10 Grass, West Warwick, RI) were used to monitor signals. Signals from the recording electrode were amplified (A-M Systems, Sequim, WA) and filtered at 0.1 Hz (low frequency) and 5 kHz (high frequency). Signals were digitized through an interface (ITC-18, HEKA Elektronik, Holliston, NY) to Axograph X software (Sydney, Australia) on a Mac Pro computer (Apple Inc, Cupertino, CA). The anesthesia level was monitored throughout the recording and rats were considered to be deeply anesthetized when the primary component of local field potential oscillations in the mPFC were between 0.5 and 1.0 Hz. Data were stored on an external hard drive for off-line analysis.

#### Histology

Recording and electrode sites were verified histologically. At the end of an electrophysiological recording, Pontamine Sky Blue was iontophoretically ejected from the recording electrode to mark the recording site (at least 20 min, −30.0 μA, constant current source, Fintronics, Orange, CT). Stimulation electrode placement was marked by iron deposits from applying 3 stimulation pulses (1 mA, 10 second duration) as previously described^52^. Immediately following the marking, rats were decapitated and their brains removed. Brains were stored in 4% paraformaldehyde with 0.05% potassium ferrocyanide in 0.1 M phosphate buffer for 12 hours and then stored in 25% sucrose in 0.1 M phosphate buffer until sectioning. Brains were cut into 60 μm sections using a freezing microtome (Leica Microsystems Inc., Wetzlar, Germany) and then Nissl stained. Recording and stimulation sites were verified by light microscopy and reconstructed using a rat brain atlas^53^.

### Golgi-Cox staining of BLA neurons

Female and male brains were rapidly removed, blocked coronally to contain all of the BLA and processed for Golgi-Cox staining according to manufacturer recommended protocol (FD Rapid GolgiStain Kit, FD Neuro Technologies, Columbia, MD). Throughout the processing, brains were stored in containers protected from light. Brain slices were collected into 20% sucrose in 0.1 M phosphate buffer (pH 7.4) at room temperature (100 μm thickness, SM 2000 R microtome, Leica). Slices were mounted on gelatinized slides and allowed to dry (25 minutes–1 hour), and rinsed in double distilled H_2_O (2 times, 4 minutes each rinse). Slides were dehydrated in 50%, 75% and 95% ethanol for 4 minutes each, then in 100% ethanol 4 times for 3 minutes each. Slides were cleared with xylene 3 times for 4 minutes each; coverslips were applied with permount and then allowed to dry for 2 weeks.

#### Neuronal reconstruction

Golgi-stained LAT and BA neurons were reconstructed using Neurolucida software (MBF Bioscience, Williston, VT) under bright field illumination with a 100x objective (Eclipse E400 microscope, Nikon, Melville, NY). Rat brain atlases were used to determine LAT and BA borders^53,54^. Only neurons that appeared to be fully impregnated were utilized. Neurons with breaks (>5 μm) in the dendrites were not included. Neurons were selected based on previously established properties of BLA neuron morphology (e.g. large cell bodies with obvious primary dendrites and spines^55^).

### Fear conditioning

Behavioral measures were obtained in a separate group of rats that were not used for electrophysiology or Golgi-Cox staining. Cued fear conditioning was performed as described previously^20^. Conditioning and extinction testing were performed four days apart for both males and females, such that the female rats were in the same estrous phase during both sessions^43^. Conditioning was conducted in chambers enclosed in sound-attenuating cabinets (Ugo Basile, Varese, Italy), with an audio speaker (Ugo Basile), a dim house light, an infrared LED light and a ceiling mounted infrared-sensitive digital camera (Fire-i, Unibrain, San Ramon, CA) in the cabinets. Conditioning consisted of 2 min habituation followed by 5 pairings of a neutral tone (10 s, 1500 Hz, 85 dB) with a co-terminating foot shock (1 s, 0.3–0.5 mA). Conditioning trials were presented at 60 s inter-trial intervals. Rats remained in the chamber for 1 min after the end of the last conditioning trial, and then were returned to their home cage. Four days later, conditioned freezing and within session acquisition of extinction were tested in a contextually distinct chamber (different wall pattern and color, odors, and flooring) to minimize contextual freezing. Testing consisted of a 2 min habituation followed by 15 trials of tone presentation (20 s, 1500 Hz, 85 dB) at a 60 s intertrial interval. No foot shock was presented during testing trials. After testing, animals were returned to their home cage. Data was collected by a computer (Dell E6500, Austin, TX) running video-tracking software (ANY-maze software, Stoelting Co, Wood Dale, IL) that detects and records freezing behavior.

### Data analysis

When possible, data was collected or analyzed blind to sex and/or treatment. Male and female control and stress groups were run in parallel.

#### Spontaneous neuronal activity

Recorded neurons were included in analyses if they were located within the BLA and if the measured action potentials were readily discernable from noise (signal to noise ratio of at least 3:1). The firing rate of BLA neurons was determined as the number of action potentials per second (Hz). The number of spontaneously active cells recorded per electrode track was measured and is reflective of the overall relative neuronal firing activity of a structure (West and Grace, 2000). Cells recorded when the primary mPFC local field potential oscillation frequency was outside a range of 0.5–1.0 Hz (possibly indicating fluctuation in depth of anesthesia) were not included in the data analysis.

#### Neuronal morphology

Neurons that had large somata with bipolar, aspiny dendrites or small somata with few, aspiny dendrites were not included in this analysis. Sholl analysis was used to measure dendritic length and number of spines within concentric circles at increasing diameters of 10 μm steps from reconstructed neurons (Sholl, 1953). Photographs of each individual LAT or BA neuron were collected using 10, 20 and 100x objectives at similar light conditions.

#### Calculation of changes across estrous cycle

To measure the direction and magnitude of changes across the estrous cycle, measures of neuronal activity, morphology and rodent behavior obtained during proestrus were normalized to measures obtained during diestrus. This was accomplished by obtaining the average value of the group in diestrus and subtracting this from each proestrus value [Individual Value_proestrus_] – [Average_diestrus_]. A value close to zero indicates little change across the estrous cycle, >0 indicates a measure that peaks in proestrus, <0 indicates a measure that peaks in diestrus.

### Statistical analysis

Statistical analysis was performed using Prism 5 software (GraphPad, La Jolla, CA). Data were compared between males and females (diestrus and proestrus combined), and compared between diestrus and proestrus females. A p < 0.05 was considered statistically significant. Data were tested for normality using the D’Agostino and Pearson omnibus normality test. If data failed the test for normality, two groups were compared by Mann-Whitney *U* test while more than 2 groups were compared by non-parametric Kruskal-Wallis test, followed by a Dunn’s multiple comparison test for significant differences between groups. If data were normally distributed, they were screened for outliers (ROUT, Q = 0.5%; removal of any outlier is listed in the Results). Two groups were compared with two-tailed unpaired t-test, while more than two groups were compared with one-way Analysis of Variance (ANOVA). When comparing across more than one factor, two-way ANOVA or two-way repeated measures ANOVA was used as appropriate. Significant results in ANOVA comparisons were followed by post hoc Holm-Sidak’s multiple comparisons test to minimize Type I errors.

## Results

### Effects of stress on estrous cyclicity

**Figure 1:**
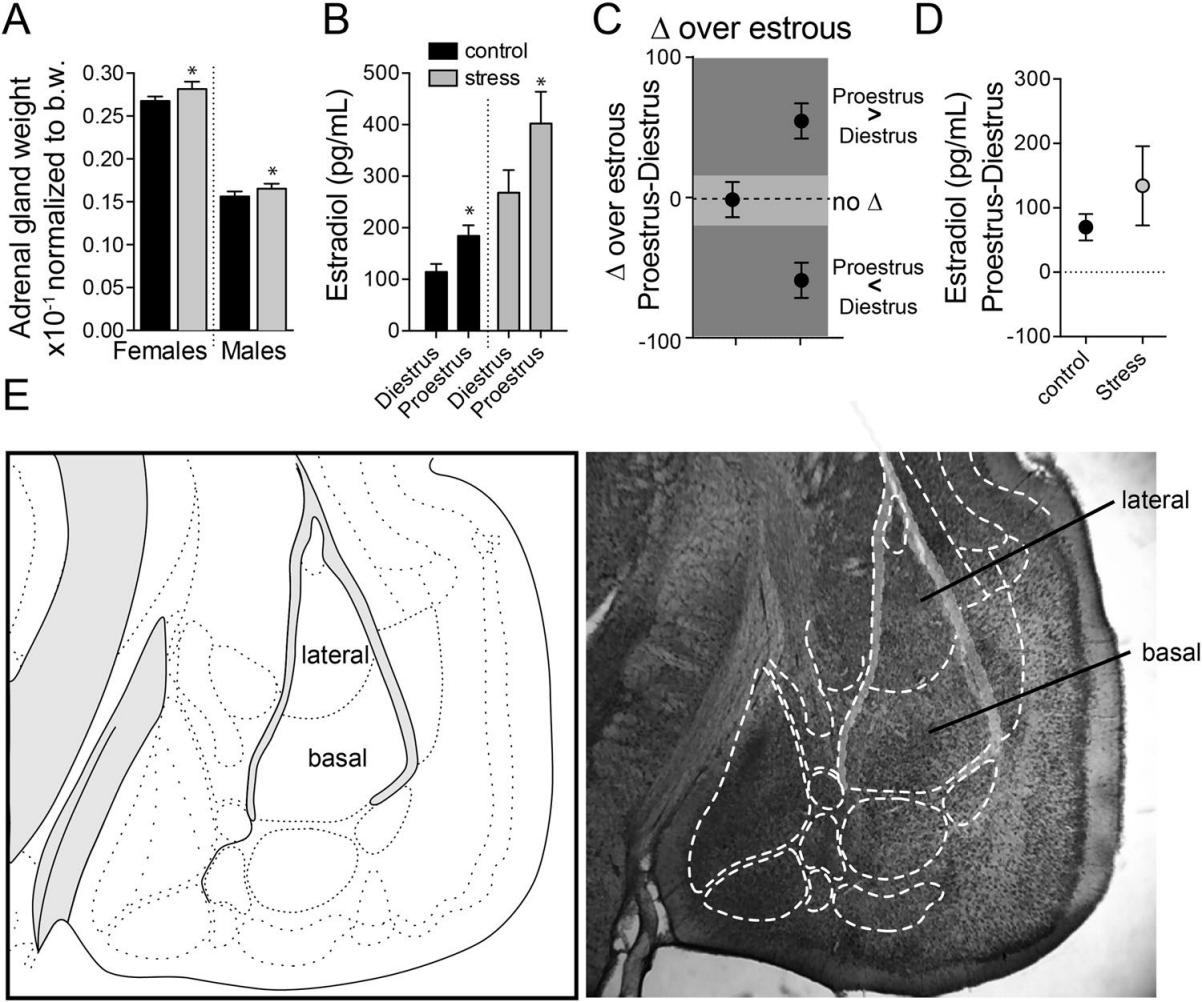
Effects of stress on endocrine measures. (**A**) Repeated stress caused an increase in adrenal gland weight. (**B**) Estradiol levels were measured from trunk blood samples and were greater in proestrus compared to diestrus. Repeated stress caused an increase of estradiol, but there was still higher estradiol in proestrus compared to diestrus. (**C**) To quantitatively compare the change that occurs between diestrus and proestrus (cyclicity), proestrus values were subtracted from the diestrus value. When presented graphically, as in this schematic example, values above 0 signify that the value was greater during proestrus, while values below 0 signify that the value was greater during diestrus. (**D**) Estradiol levels were greater during proestrus (value > 0), and the degree of cyclicity was not different between control and stress. (**E**) A representation of nucleus borders (left; modified from^97^), and borders overlaid on a Nissl stained section of tissue (right). All values mean ± s.e.m. *p < 0.05 Holm-Sidak’s multiple comparisons test after 2-way ANOVA.

Repeated restraint stress increased adrenal gland weight in male and female rats (main effect of stress, F(1,140) = 5.948, p = 0.0160, two-way ANOVA; p < 0.05 Holm-Sidak’s multiple comparisons test, n = 65 control female, n = 31 stress female, n = 26 control male, n = 22 stress male), indicating that this was an effective stressor in both sexes. Repeated restraint stress did not disrupt the regular estrous cyclicity of the female rats. Control and stress rats showed consistent 4 day cycles as assessed by vaginal lavage, and uterine size that matched diestrus and proestrus states. Estradiol levels were higher during proestrus (Fig. 1; main effect of phase, F(1,17) = 9.627, p = 0.0065, two-way ANOVA, n = 4–7 rats/group), and repeated stress increased estradiol levels (main effect of stress, F(1,17) = 31.87, p < 0.0001), but the change in estradiol levels across estrous was similar in control and stress groups (Fig. 1; [average estradiol]_proestrus_ − [estradiol]_diestrus_; p = 0.252, t = 1.224, df = 9, two-tailed t-test).

### *In vivo* extracellular recordings

#### Firing rate of neurons

**Figure 2:**
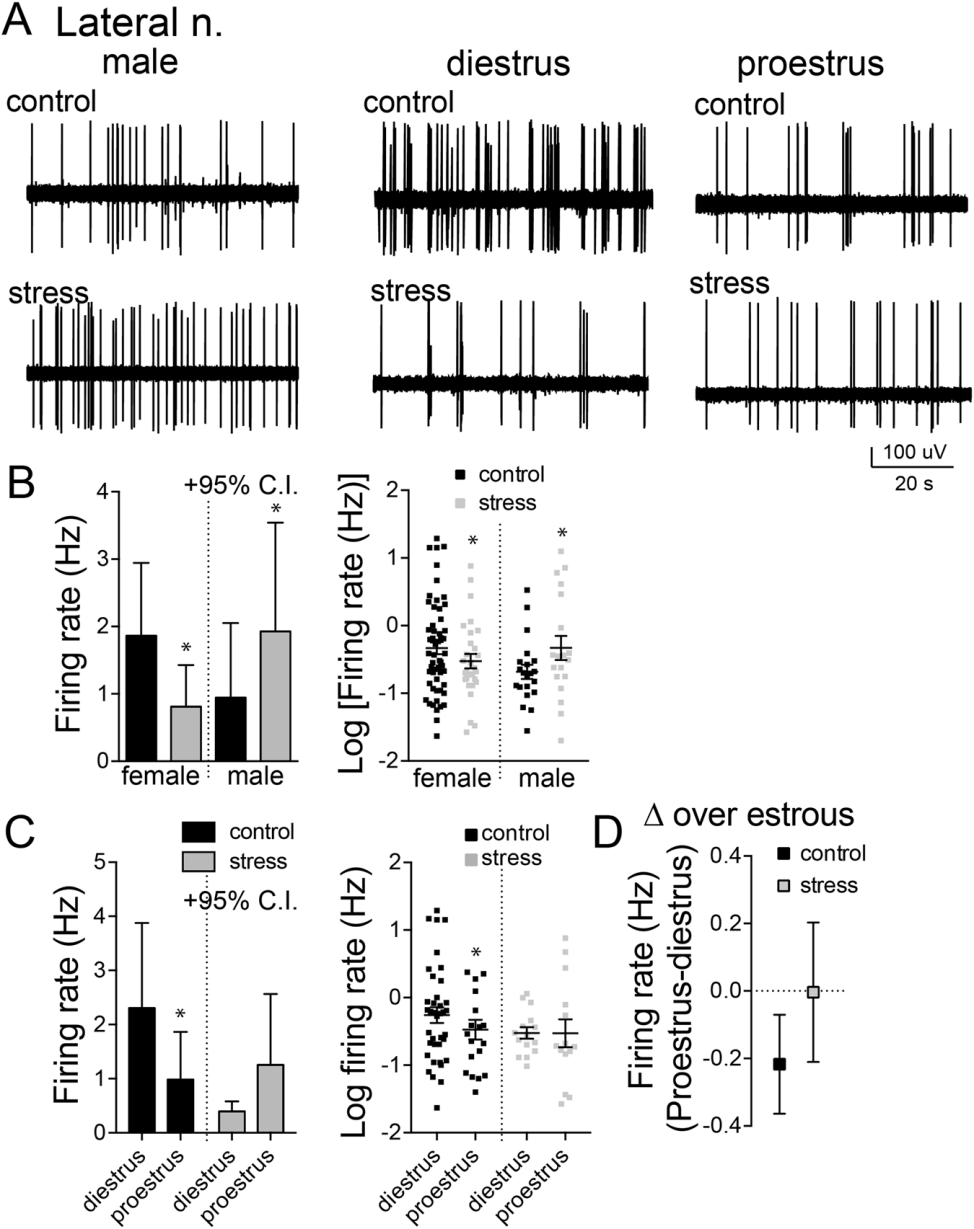
Repeated stress decreased firing rate and cyclicity of LAT neurons in females. (**A**) Single LAT neurons were recorded from male and female rats. (**B**) The firing rate of LAT neurons was decreased after repeated stress in female rats, but was increased by repeated stress in male rats (left, mean + 95% confidence interval (C.I.); right, log10 transformed data mean ± s.e.m.). (**C**) The firing rate of LAT neurons was lower during proestrus in control rats. But there was no difference between diestrus and proestrus after repeated stress (left, mean + 95% C.I.; right, log10 transformed data mean ± s.e.m.). (**D**) The cyclicity index was decreased after repeated stress. *p < 0.05 Holm-Sidak’s multiple comparisons test after 2-way ANOVA.

Recordings from LAT and BA nuclei of control rats (Fig. 1; 39 female rats and 14 male rats) were compared with recordings from rats exposed to repeated stress (Fig. 2A; 24 female rats and 12 male rats). Repeated stress increased the activity of BLA neurons in male rats (Table 1; data not Gaussian, D’Agostino & Pearson omnibus normality test, p < 0.0001; significant difference between medians, p = 0.0154, *H*(3) = 10.41, Kruskal-Wallis test; p < 0.05 Dunn’s multiple comparison post hoc test). In contrast and contrary to our expectation, repeated stress decreased the firing of BLA neurons in female rats (Table 1; p < 0.05 Dunn’s multiple comparison post hoc test). Because this data is a key finding, we normalized data into a Gaussian distribution (Log10 transformation, D’Agostino & Pearson omnibus normality test, p > 0.05) and compared groups with more powerful parametric tests to decrease the risk of an erroneous false positive. This analysis uncovered a significant interaction between stress and sex on BLA neuron firing rate (sex × stress interaction, p = 0.0112, F(1,255) = 6.527, two-way ANOVA, 1 outlier removed from LAT control male group), confirming that repeated stress had different effects on BLA neuron firing rate in females and males.

**Table 1:** The mean firing rate of neurons recorded *in vivo*.

Sex	Tx	BLA		LAT		BA		
		mean	n	mean	n	mean	n	Rats
Male	*control*	0.67 ± 0.25	42					14
	*stress*	1.72 ± 0.46	34					12
Female	*control*	2.26 ± 0.37	113	1.87 ± 0.54	57	2.66 ± 0.54	56	39
	*stress*	1.23 ± 0.22	70	0.81 ± 0.30	29	1.53 ± 0.29	41	24
diestrus	*control*			2.31 ± 0.77	38	2.26 ± 0.63	32	23
	*stress*			0.40 ± 0.09	15	1.19 ± 0.34	29	15
proestrus	*control*			0.99 ± 0.42	19	3.19 ± 0.96	24	16
	*stress*			1.26 ± 0.61	14	2.35 ± 0.54	12	9

The mean ± s.e.m. is presented along with the number of neurons. Because multiple neurons were usually recorded, the number of rats is also shown here.

#### Relative number of active neurons

In addition to impacting neuronal firing, repeated stress may impact the number of active neurons in the BLA. To examine this possibility, we measured the number of spontaneously active neurons encountered and recorded in the first electrode penetration of each animal. The number of active neurons per electrode track in females (control: 3.00 ± 0.25, 39 rats; stress: 3.15 ± 0.31, 24 rats) was similar to males (control: 2.63 ± 0.30, 12 rats; stress: 3.14 ± 0.42, 14 rats; main effect of sex, p = 0.557, two-way ANOVA, F(1,90) = 0.347; data not depicted), and repeated stress did not significantly impact the number of active neurons (main effect of stress, p = 0.316, F(1,90) = 1.018; stress × sex interaction p = 0.588, F(1,90) = 0.295, two-way ANOVA; data not depicted).

#### Firing rate of LAT and BA neurons

**Figure 3:**
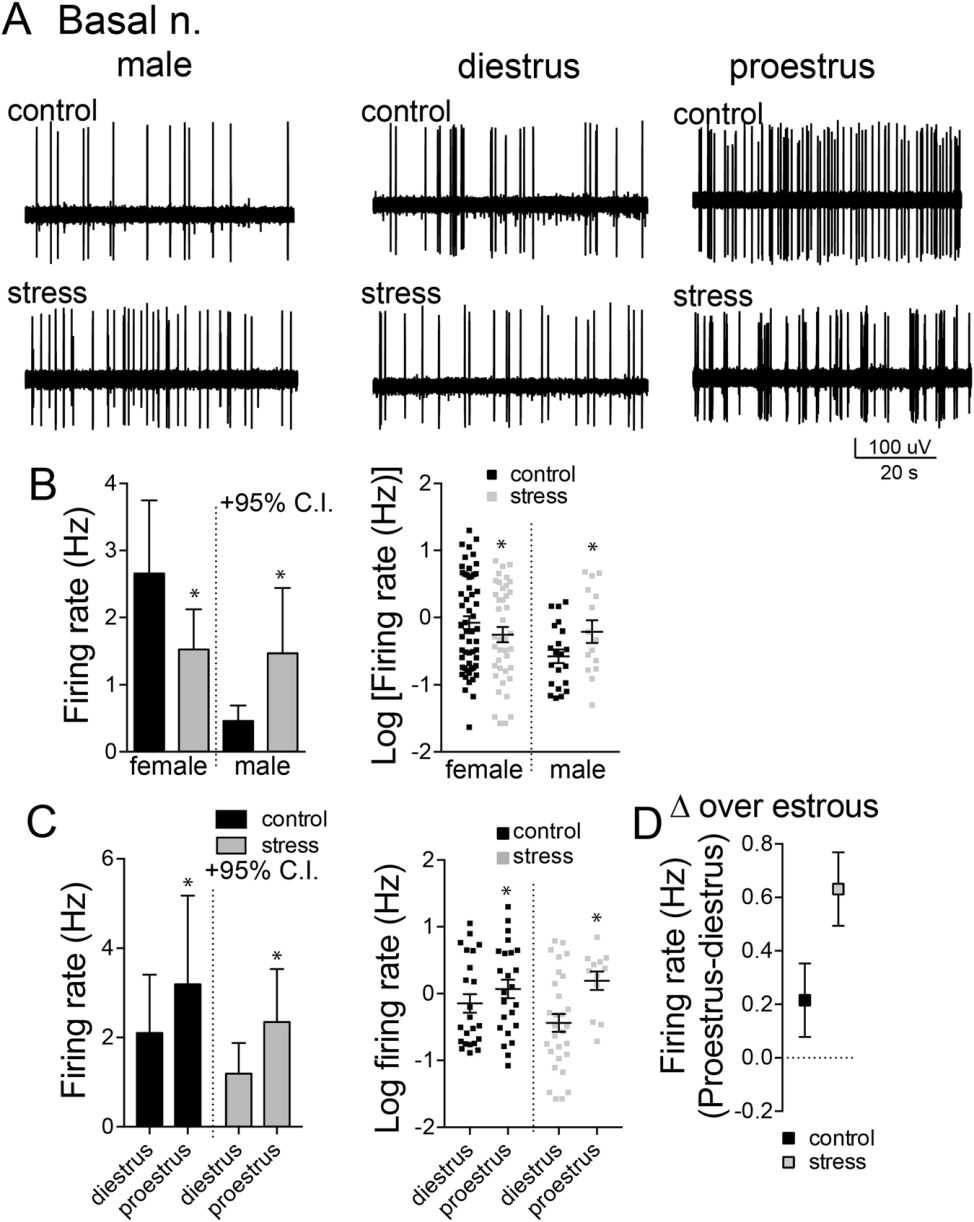
Repeated stress decreased firing rate and increased cyclicity of BA neurons in females. (**A**) Single BA neurons were recorded from male and female rats. (**B**) The firing rate of BA neurons was decreased after repeated stress in female rats, but was increased by repeated stress in male rats (left, mean + 95% C.I.; right, log10 transformed data mean ± s.e.m.). (**C**) The firing rate of BA neurons was greater in proestrus compared to diestrus in control rats and after repeated stress (left, mean + 95% C.I.; right, log10 transformed data mean ± s.e.m.). (**D**) This sensitivity to estrous was increased after repeated stress, reflected as increase in the cyclicity index after repeated stress (mean ± s.e.m.). *p < 0.05 Holm-Sidak’s multiple comparisons test after 2-way ANOVA.

The LAT and BA nuclei of the BLA are functionally distinct in the afferents they receive and their roles in behavior. We therefore examined whether restraint stress had a different impact on neuronal firing rate in the LAT and BA between female and male groups. Stress increased the firing rate of LAT neurons in males (Fig. 2B, Table 1; sex × stress interaction, p = 0.0322, F(1,122) = 4.694; main effect of stress, p = 0.7621, F(1,122) = 0.09205, two-way ANOVA on transformed data, 1 outlier removed from LAT control male group; males, p < 0.05 Holm-Sidak’s multiple comparisons test) but significantly decreased the firing of LAT neurons in female rats (females, p < 0.05, Holm-Sidak’s multiple comparisons test). In the BA, repeated stress significantly increased the firing of neurons in the BA of male rats (Fig. 3B, Table 1; sex × treatment interaction, p = 0.045, F(1,129) = 4.111, two-way ANOVA on transformed data; males, p < 0.05, Holm-Sidak’s multiple comparisons test) but significantly decreased the firing of BA neurons in female rats (females, p < 0.05, Holm-Sidak’s multiple comparisons test). Thus, repeated stress had opposite effects in males and females on both LAT and BA neuron firing.

#### Firing rate across estrous

We previously found that the firing of BLA neurons in females fluctuates across the estrous cycle, whereby the firing of LAT neurons is higher in diestrus, and the firing of BA neurons is higher during proestrus^43^. That pattern was replicated here (Figs 2C and 3C; cycle × nucleus interaction, p = 0.026, F(1,109) = 5.098, two-way ANOVA on transformed data). To test whether stress impacted this pattern that shifts across estrous, a single measure of change across estrous was quantified (Methods; [firing rate]_proestrus_ – [firing rate]_diestrus_). In females exposed to stress, the pattern was disrupted such that there was no change in LAT activity across estrous (Fig. 2D; main effect of stress, p = 0.049, F(1,65) = 4.025, two-way ANOVA on transformed data; not significantly different than 0 after stress, p > 0.05, one sample t-test), but the pattern of change of BA activity across estrous was even greater in rats exposed to stress (Fig. 3D; p < 0.05, Holm-Sidak’s multiple comparisons test).

#### Firing pattern

**Figure 4:**
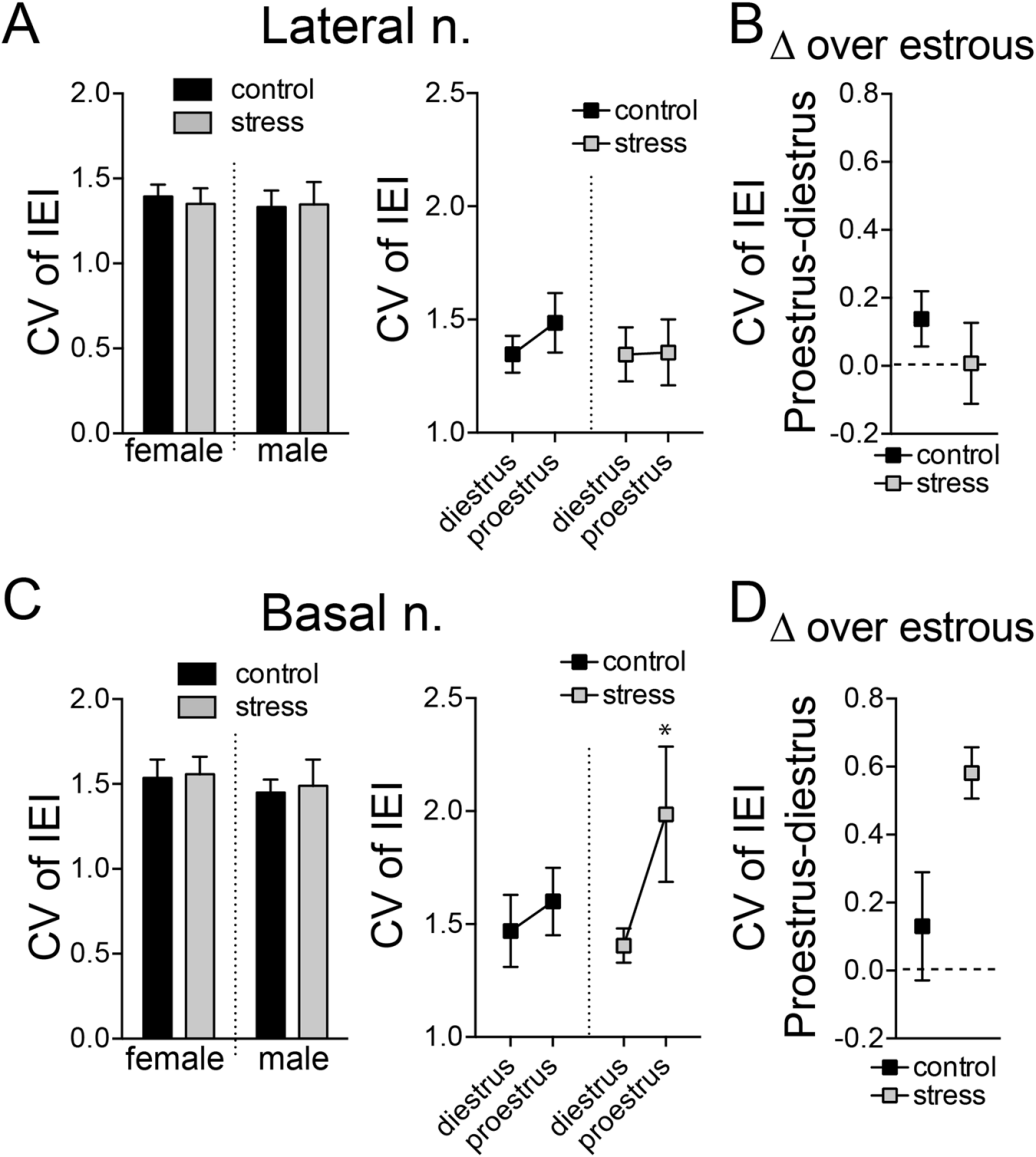
The variability of neuronal firing across the cycle was altered by stress. The coefficient of neuronal firing (CV) was measured as an index of firing variability. (**A**) The overall CV of LAT neuron firing was not impacted by repeated stress (left). The CV was not significantly different between diestrus and proestrus in control rats or after repeated stress (right). (**B**) The cyclicity index for CV of LAT neurons was similar between control and stress groups. (**C**) The overall CV of BA neuron firing was not impacted by repeated stress (left). While CV of BA neurons was similar between proestrus and diestrus in control groups, after repeated stress the CV was significantly higher in proestrus compared to diestrus (right). (**D**) The cyclicity index for CV of BA neurons was increased by repeated stress. All data mean ± s.e.m. *p < 0.05 Holm-Sidak’s multiple comparisons test after 2-way ANOVA.

The firing of BLA neurons is driven by synaptic inputs. Variability of neuronal firing pattern can reflect the excitatory-inhibitory balance of inputs and network state of that region^56–60^. One way to quantify this is by coefficient of variation (CV) of the interspike-interval of firing for each neuron. Stress did not impact the overall CV of LAT neuron firing (Fig. 4A; stress x sex interaction, p = 0.767, F(1,122) = 0.0880; main effect of stress, p = 0.89, F(1,122) = 0.021) or BA neuron firing BA (Fig. 4B; stress × sex interaction, p = 0.942, F(1,129) = 0.005; main effect of stress, p = 0.81, F(1,129) = 0.057, two-way ANOVA).

While stress did not impact overall CV, it may impact the pattern of CV changes across the estrous cycle. In the LAT, CVs were not different between the cycle stages (Fig. 4C; phase × stress interaction, p = 0.591, F(1,82) = 0.292; main effect of phase, p = 0.547, F(1,82) = 0.376, two-way ANOVA). In the BA, while there was no difference of CV across the estrous cycle in controls, a difference of CV between cycle stages emerged after stress (Fig. 4C; main effect of phase, p = 0.029, F(1,81) = 4.957, two-way ANOVA; phase × stress interaction, p = 0.1622, F(1,81) = 1.981, two-way ANOVA) with increased CV during proestrus (p < 0.05, Holm-Sidak’s multiple comparisons test). The change of CV across estrous ([CV]_proestrus_ − [CV]_diestrus_) was significantly increased in BA by stress (stress × nucleus interaction, p = 0.0105, F(1,100) = 6.799, two-way ANOVA; BA, p < 0.05, Holm-Sidak’s multiple comparisons test), but not in LAT (LAT, p > 0.05, Holm-Sidak’s multiple comparisons test). These results indicate that, while overall CV of LAT or BA neuron firing was not sensitive to stress, the fluctuation of BA neuron CV across estrous was sensitive to stress. Because CV can reflect synaptic input effects on firing, this sensitivity suggests that stress impacts whether inputs to BA neurons shift over the estrous cycle.

### Golgi-Cox staining of BLA neurons

**Figure 5:**
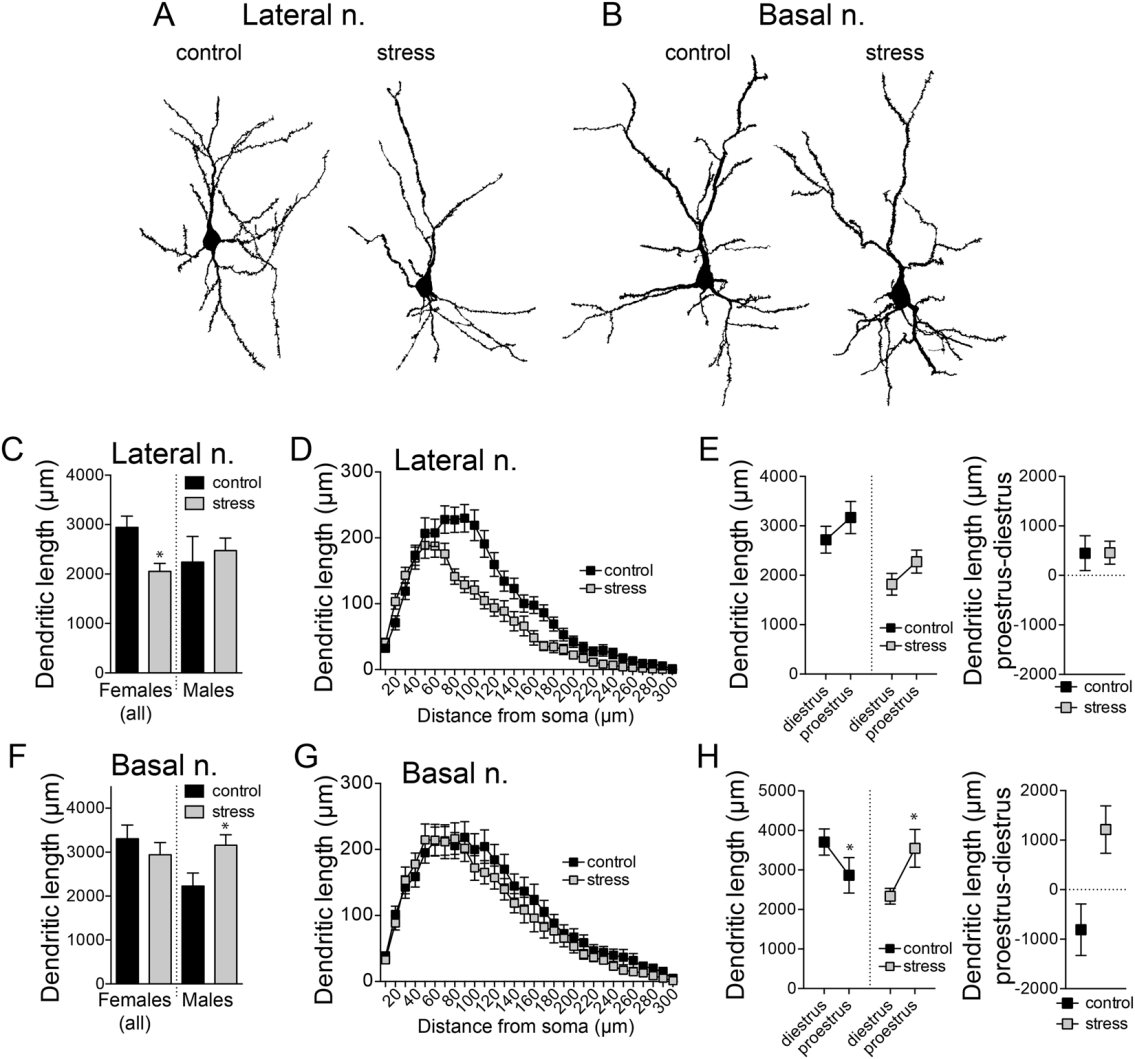
Unique effects of repeated stress on dendritic length of BLA neurons in females. BLA neurons were Golgi-stained and reconstructed. (**A**) Examples of reconstructed LAT neurons after control (left) or stress (right). (**B**) Examples of reconstructed BA neurons after control (left) or stress (right). (**C**) Repeated stress decreased the dendritic length of LAT neurons in females, but had no significant effect in males. (**D**) Repeated stress significantly decreased dendritic length, particularly at intermediate distances from the soma, as indicated by Sholl analysis. (**E**) Dendritic length of LAT neurons was similar between diestrus and proestrus in control and stress groups (left). Repeated stress did not significantly impact the sensitivity of LAT neurons to estrous (right). (**F**) Repeated stress did not significantly impact the overall dendritic length of BA neurons in females, but increased it in males. (**G**) The distribution of the dendritic length across the dendritic tree was similar in control and stress groups, as indicated by Sholl analysis. (**H**) Dendritic length was shorter during proestrus compared to diestrus in control rats. But after repeated stress, dendritic length was shorter during diestrus, and the cyclicity index of BA neurons was flipped (right). All data mean ± s.e.m. *p < 0.05 Holm-Sidak’s multiple comparisons test after 2-way ANOVA.

Excitatory inputs drive BLA neuronal firing and are sensitive to repeated stress in males^61–63^. The dendrites are a key site for synaptic inputs. As the majority of excitatory inputs into the BLA synapse on the spines of pyramidal neurons^64–66^, spine number is used as an index for excitatory input and is sensitive to stress in males (Vyas *et al*., 2002, #5370; Padival *et al*., 2013a, 2013b). Dendritic length is sensitive to stress in males, and can play a functional role in neuronal responsiveness. To investigate changes in dendritic spines and morphology, Golgi-impregnated BLA pyramidal neurons were reconstructed for morphological analysis (Fig. 5A,B).

*Dendritic length*. In agreement with our previous studies, stress had no significant effect on dendritic length of LAT neurons in male rats (Fig. 5C; p = 0.6903, t = 0.4030, df = 26; N = 14 control, N = 14 stress, two-tailed unpaired t-test), and increased the dendritic length of BA neurons in male rats (Fig. 5F; p = 0.0183, t = 2.505, df = 28; N = 16 control, N = 14 stress, two-tailed unpaired t-test).

Very different effects of stress were found in females. Repeated stress decreased dendritic length of LAT neurons in females (Fig. 5C,D; stress × distance interaction, p < 0.0001, F(29,1769) = 5.031; main effect of stress, p = 0.0023, F(1,61) = 10.09; stress × phase interaction, p = 0.9853, F(1,59) = 0.000, two-way RM-ANOVA). The dendritic length was not significantly different between diestrus and proestrus (main effect of phase, p = 0.1086, F(1,59) = 2.654, two-way RM-ANOVA). Stress did not impact the pattern of dendritic length across estrous ([Length_proestrus_] − [Length_diestrus_]; Fig. 5E; p = 0.9806, t = 0.02458, df = 30, two-tailed unpaired t-test).

In contrast, while stress had minimal effect on dendritic length of BA neurons in females (Fig. 5F,G; stress × distance interaction, p = 0.9150, F(29,1798) = 0.6619; main effect of stress, p = 0.3873, F(1,62) = 0.7582, two-way RM-ANOVA), the sensitivity of dendritic length to the estrous cycle was impacted by stress (Fig. 5H; stress × phase interaction p = 0.0149, F(1,60) = 6.287; main effect of stress p = 0.3706, F(1,60) = 0.8137, two-way ANOVA). BA neuron dendritic length in controls was higher during diestrus, but this was flipped after stress, such that BA dendritic length was higher during proestrus (Fig. 5H; p = 0.0076, t = 2.860, df = 30, two-tailed unpaired t-test). These results point to unique effects of stress on LAT and BA neurons from female rats, and consistently demonstrate an effect of stress on the sensitivity of LAT and BA neurons to the estrous cycle.

#### Dendritic spines

**Figure 6:**
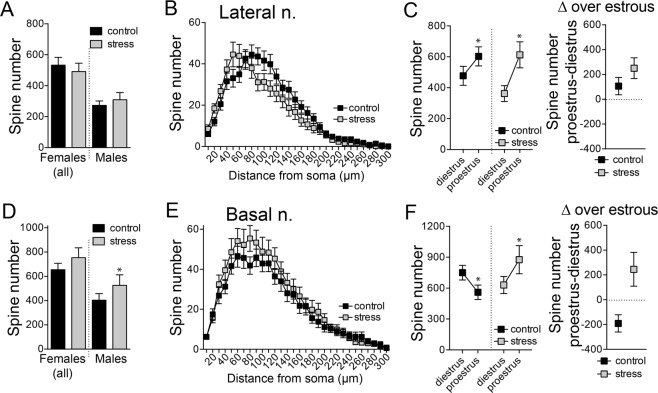
Repeated stress altered the estrous cyclicity of dendritic spines in BA neurons. The number of spines was quantified from reconstructed neurons. (**A**) Repeated stress did not significantly change the number of spines counted in LAT neurons. (**B**) There was a small but significant shift in the distribution of dendritic spines after repeated stress, toward more proximal loci, as indicated by Sholl analysis. (**C**) The number of LAT neuron spines was greater in proestrus compared to diestrus, in both control and stress groups (left). Repeated stress did not significantly impact the sensitivity of LAT neurons to the estrous cycle (right). (**D**) Repeated stress did not significantly impact the number or distribution of spines in BA neurons from females, but increased the number of spines in BA neurons from males. (**E**) There was a trend towards an increase in the number of spines in BA neurons from females at proximal sites, as indicated by Sholl analysis. (**F**) The number of BA neuron spines was greater during diestrus compared to proestrus in control rats, however, the number of spines was greater during proestrus in the stress group (left). Repeated stress flipped the estrous sensitivity of BA neurons and flipped the cyclicity index of BA neurons (right). All data mean ± s.e.m. *p < 0.05 Holm-Sidak’s multiple comparisons test after 2-way ANOVA.

In LAT neurons, stress did not significantly change the number of spines (Fig. 6A), but caused a proximal shift in the distribution of dendritic spines in females (Fig. 6B; stress × distance interaction, p < 0.0001, F(29,1769) = 2.689; main effect of stress, p = 0.4267, F(1,61) = 0.6404, two-way RM-ANOVA, N = 32 neurons control, 31 neurons stress). The number of spines on LAT neurons was higher on proestrus compared to diestrus (Fig. 6C; main effect of phase, p = 0.0129, F(1,59) = 6.575, two-way RM-ANOVA), a pattern that was not significantly impacted by stress (main effect of stress p = 0.3750, F(1,59) = 0.7990, stress × phase interaction p = 0.3033, F(1,59) = 1.078, two-way ANOVA). Similarly, the index for change of LAT spine number in proestrus and diestrus (Spines_proestrus_] − [Spines_diestrus_]) was not significantly impacted by stress (p = 0.1958, t = 1.323, df = 30, two-tailed unpaired t-test).

In BA neurons, there was no significant effect of stress on spine number in females (Fig. 6D; stress × distance interaction, p = 0.7754, F(29,1798) = 0.7931; main effect of stress, p = 0.3147, F(1,62) = 1.027, two-way RM-ANOVA, N = 32 neurons control, 32 neurons stress). The number of BA spines changed across the estrous cycle and was higher in diestrus (main effect of phase, p = 0.0359, F(1,30) = 4.824, two-way repeated measures ANOVA). However, stress flipped the effects of estrous cycle on spine number in the BA so that it was higher in proestrus after stress (Fig. 6E,F; stress × phase interaction, p = 0.0237, F(1,60) = 5.387, main effect of phase, p = 0.7716, F(1,60) = 0.085, two-way ANOVA; p < 0.05, Holm-Sidak’s multiple comparisons test). When measured directly, the index of change across the estrous cycle was significantly different, and opposite direction, between control and stress groups (p = 0.0078, t = 2.853, df = 30, two-tailed unpaired t-test; control peak in diestrus, stress peak in proestrus).

In stark contrast to females, stress increased the number of spines in BA neurons from males (stress × distance interaction, p < 0.0001, F(29,812) = 2.520, two-way RM-ANOVA, N = 16 control, N = 14 stress), similar to previous findings^38,42,62^. Stress did not significantly increase the number of spines in LAT neurons from males, though there was a strong trend (stress × distance interaction, p = 0.0696, F(29,754) = 1.425, two-way RM-ANOVA, N = 14 control, N = 14 stress).

### Effects of stress on fear conditioning

Fear conditioning is strongly dependent on the BLA, and has been shown to be sensitive to stress. Three aspects of fear conditioning were examined, acquisition of conditioned freezing, initial expression of conditioned freezing, and acquisition of extinction of conditioned freezing. Acquisition of conditioned freezing was measured on the conditioning day. During tests of conditioned fear after 4 days, the initial conditioned fear response and eventual acquisition of extinction of the conditioned fear response were quantified. To measure the conditioned fear response, the first three conditioned freezing tone trials on the testing day (before significant extinction of response is observed) were collapsed and compared across groups. Acquisition of extinction was measured as the last 10 trials (trials 5–15).

#### Fear conditioning

**Figure 7:**
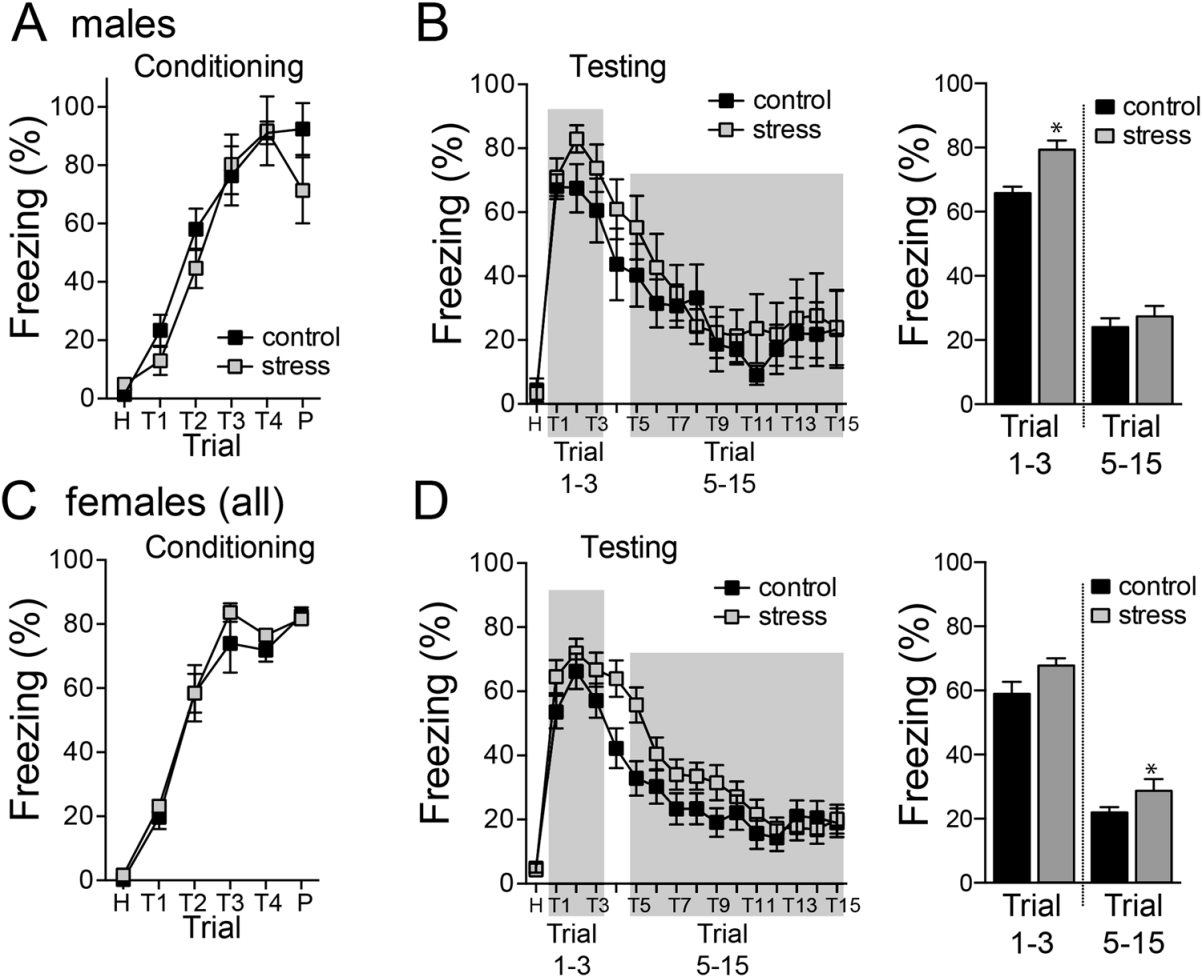
Effects of repeated stress on conditioned fear. (**A**) Repeated stress did not significantly impact freezing during fear conditioning in males. (**B**) The initial expression of conditioned fear (trials 1–3) and the acquisition of extinction (trials 5–15) were both measured. Repeated stress increased the initial expression of conditioned fear in male rats (trials 1–3), but not the acquisition of extinction (trials 5–15). (**C**) Repeated stress did not significantly impact freezing during fear conditioning in females. (**D**) Repeated stress did increase the initial expression of conditioned fear in female rats (trials 1–3), but decreased the acquisition of extinction (trials 5–15). All data mean ± s.e.m. *p < 0.05 Holm-Sidak’s multiple comparisons test after 2-way ANOVA.

In males, there was no effect of repeated stress on acquisition of conditioned fear (Fig. 7A; main effect of stress, p = 0.335, F(1,15) = 0.992; stress × trial interaction, p = 0.408, F(5,75) = 1.027, two-way repeated measures ANOVA; control n = 8 rats, stress n = 9 rats). Repeated stress increased conditioned freezing on the test day (Fig. 7B; trials 1–3, main effect of stress, p = 0.0310, F(1,16) = 5.593, two-way repeated measures ANOVA). But there was no effect of repeated stress on the acquisition of extinction (trials 5–15, main effect of stress, p = 0.4672, F(1,16) = 0.555; stress × trial interaction, p = 0.981, F(10,160) = 0.299, two-way repeated measures ANOVA).

In females, there was no effect of repeated stress on acquisition of conditioned fear (Fig. 7C; main effect of stress, p = 0.550, F(1,56) = 0.361; stress × trial interaction, p = 0.860, F(5,280) = 0.383, two-way repeated measures ANOVA; control n = 8 rats, stress n = 9 rats). There was a strong trend towards an effect of repeated stress on the conditioned freezing on the test day (Fig. 7D; trials 1–3, main effect of stress, p = 0.0577, F(1,56) = 3.755, two-way repeated measures ANOVA). However, repeated stress decreased the acquisition of extinction (Fig. 7D; trials 5–15, main effect of stress, p = 0.130, F(1,56) = 2.358; stress × trial interaction, p = 0.0259, F(10,560) = 2.060, two-way repeated measures ANOVA). This points to unique effects of stress on conditioned fear in females.

**Figure 8:**
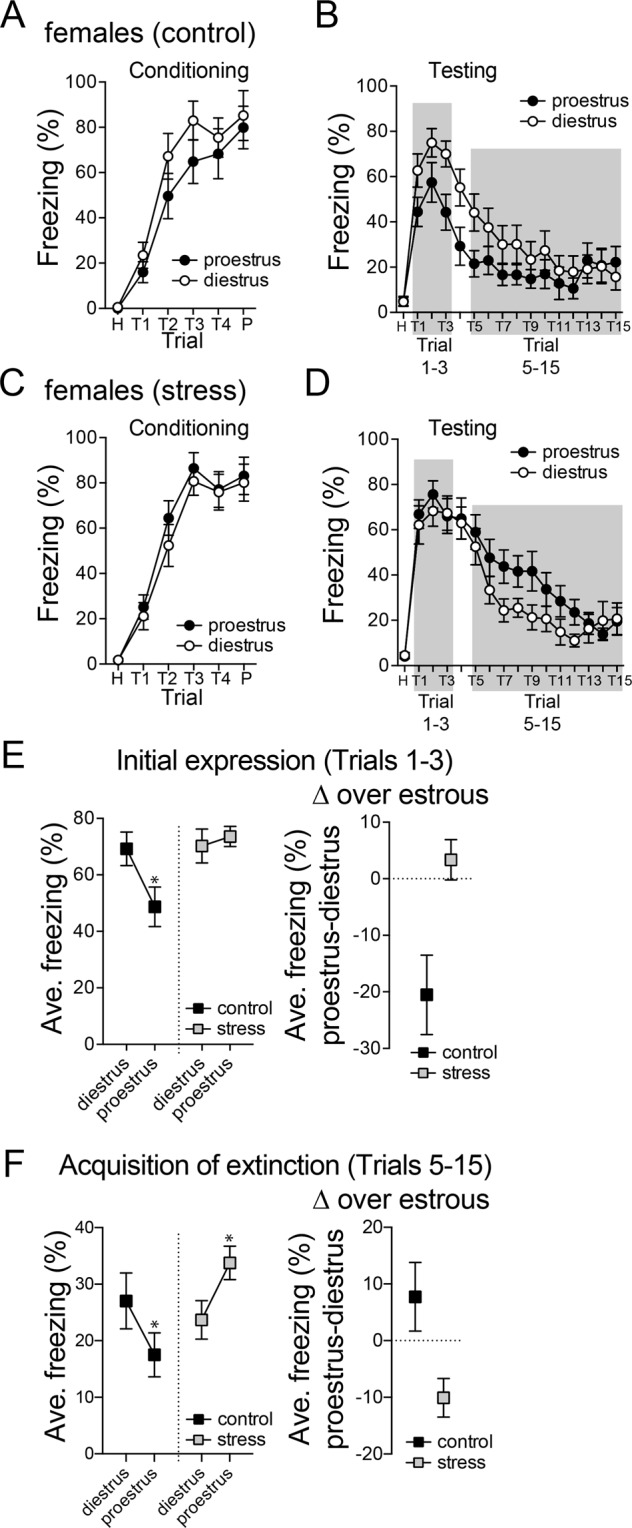
Repeated stress disrupts the estrous cyclicity of conditioned fear and extinction. (**A**) In controls, there was no significant difference in freezing during fear conditioning between diestrus and proestrus. (**B**). A significant difference in conditioned freezing during testing was observed between diestrus and proestrus in control rats, with greater freezing during diestrus. (**C**) After repeated stress, there was no significant difference in freezing during fear conditioning across the estrous cycle. (**D**) A significant difference in conditioned freezing during testing was observed across the estrous cycle after repeated stress, with greater freezing during proestrus. (**E**) The initial conditioned freezing during testing (trials 1–3) was compared. Initial conditioned freezing was sensitive to the estrous cycle in controls, with greater freezing during diestrus. After repeated stress, this sensitivity was absent and freezing was high during diestrus and proestrus. The cyclicity of the initial conditioned response (trials 1–3) was decreased after repeated stress (right). (**F**) The acquisition of with-in session extinction (Trials 5–15) was compared. Freezing during extinction trials was sensitive to the estrous cycle in control groups, with greater freezing during diestrus. Freezing during extinction trials was also sensitive to the estrous cycle in stress groups, but with higher freezing during proestrus, and overall higher levels of freezing than controls. The cyclicity of extinction freezing was reversed between control and stress groups (right). All data mean ± s.e.m. *p < 0.05 Holm-Sidak’s multiple comparisons test after 2-way ANOVA.

We then tested if conditioned freezing differs between diestrus and proestrus, and if this is disrupted by repeated stress. There was no significant difference in acquisition of conditioned fear between diestrus and proestrus females, measured as freezing on the day of conditioning in either control rats (Fig. 8A; main effect of phase, p = 0.2497, F(1,28) = 1.382; phase × trial interaction, p = 0.7390, F(5,140) = 0.5488, two-way RM-ANOVA, control N = 15 proestrus, N = 15 diestrus) or after repeated stress (Fig. 8C; main effect of phase, p = 0.4804, F(1,26) = 0.5126; phase × trial interaction, p = 0.9225, F(5,130) = 0.2816, two-way RM-ANOVA, repeated stress N = 14 proestrus, N = 14 diestrus). Previous work demonstrates that the conditioned fear response shifts across estrous^43^. This was replicated here, with greater conditioned freezing during diestrus compared to proestrus (Fig. 8B; phase × trial interaction, p = 0.0023, F(15,420) = 2.409, two-way RM-ANOVA, control N = 15 proestrus, N = 15 diestrus). The initial expression of conditioned freezing (trials 1–3) was higher during diestrus compared to proestrus (Fig. 8E; main effect of phase, p = 0.0335, F(1,28) = 5.001, two-way RM-ANOVA, control N = 14 proestrus, N = 14 diestrus). However, repeated stress disrupted this, and there was no significant difference between diestrus and proestrus (Fig. 8E; main effect of phase, p = 0.6881, F(1,26) = 0.1648; phase × trial interaction, p = 0.5876, F(2,52) = 0.5372, two-way RM-ANOVA, control N = 15 proestrus, N = 15 diestrus). This was directly compared with similar results, repeated stress dampened the shift of conditioned freezing that usually occurs between diestrus and proestrus (Fig. 8E, right; [Freezing_proestrus_] − [Freezing_diestrus_]; p = 0.0063, t = 2.970, df = 27, two-tailed unpaired t-test); instead, after repeated stress, conditioned freezing (trials 1–3) was high in both diestrus and proestrus.

#### Fear extinction

Previous studies found that fear extinction also changes across the estrous cycle. We replicated previous findings that acquisition of fear extinction differs between diestrus and proestrus in control rats (Fig. 8F; phase × trial interaction, p = 0.0305, F(10,280) = 2.03, two-way RM-ANOVA), measured as the average within session freezing across extinction trials (trials 5–15). After repeated stress, there was still a significant difference of acquisition of extinction between diestrus and proestrus (Fig. 8F; main effect of phase, p = 0.0338, F(1,26) = 5.023; phase × trial interaction, p = 0.3837, F(10,260) = 1.073, two-way RM-ANOVA; stress × phase interaction, p = 0.0436, F(1,54) = 4.268, two-way ANOVA). However, repeated stress flipped the sensitivity of extinction to the estrous cycle, such that there was slower extinction in diestrus in control rats, but slower extinction in proestrus after repeated stress (Fig. 8F, right; p = 0.0180, t = 2.519, df = 27, two-tailed unpaired t-test). Repeated stress decreased the acquisition of fear extinction in female rats such that freezing during extinction was high in diestrus and proestrus relative to the control group.

## Discussion

**Figure 9:**
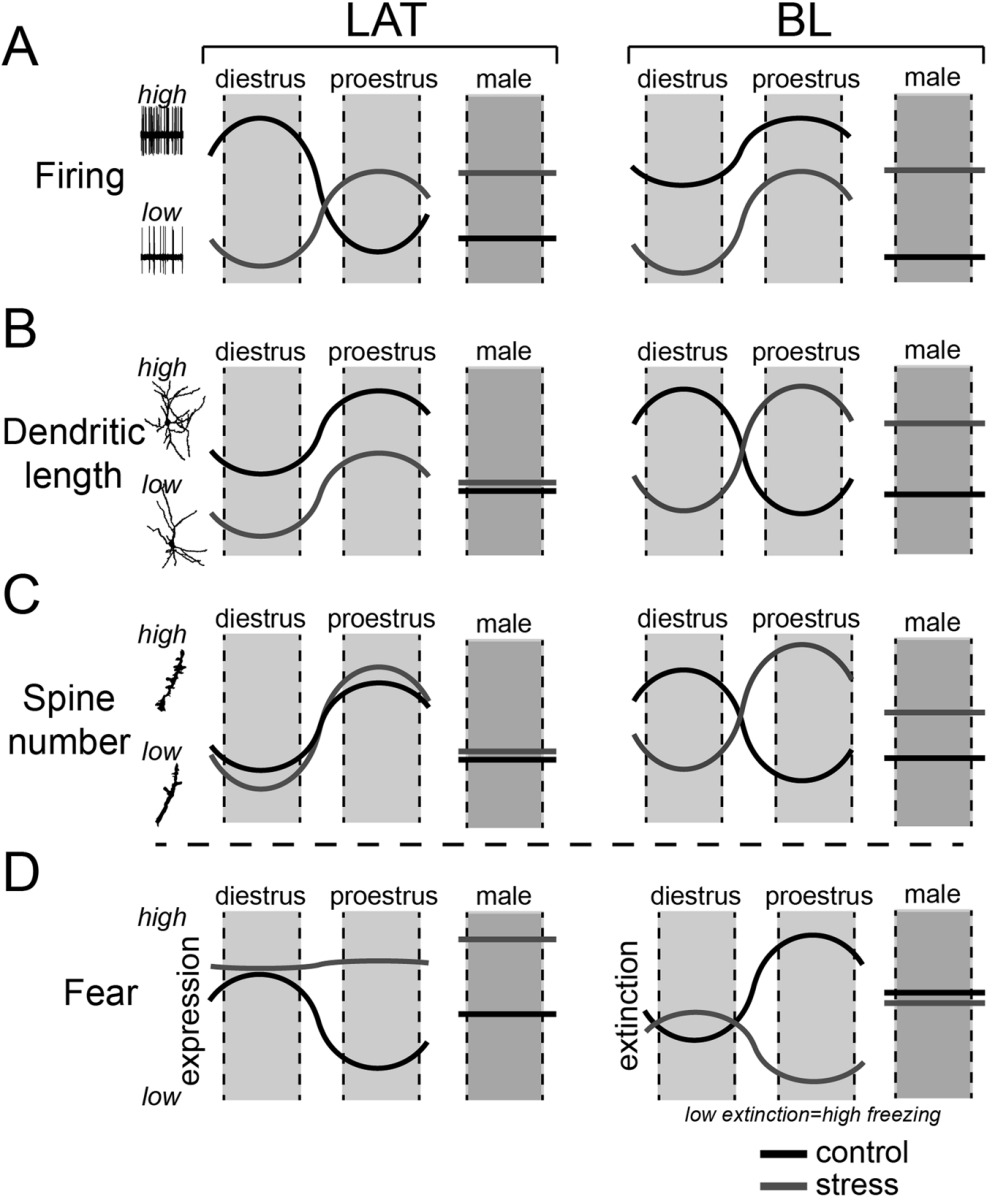
Summary of the effects of stress on BLA neurons and fear behaviors. The effects of stress on neuronal properties and fear conditioning behaviors are represented across the estrous cycle. The pattern in control animals (black line) often shows a shift between diestrus and proestrus (left to right). The pattern after repeated stress (grey line) is represented similarly. For comparison, control and stress male groups are summarized (right). These summaries are presented for LAT (left column) and BA (right column). (**A**) The firing activity of LAT neurons is higher in diestrus than proestrus, and this pattern is flipped after repeated stress (with an overall lower firing if collapsed across estrous). In males, a low LAT firing activity is greatly increased after repeated stress. The firing activity of BA neurons is higher is proestrus than diestrus. While this pattern is maintained after repeated stress, the firing of BA neurons is decreased across estrous. In males, a low BA firing activity is greatly increased after repeated stress. (**B**) Dendritic length of LAT neurons shifts across estrous, with greater length during proestrus. This pattern is maintained after repeated stress, but with decreased dendritic length across estrous. In males, the dendritic length of LAT neurons is not greatly changed after repeated stress. Dendritic length of BA neurons shifts across estrous in a manner opposite to LAT, with greater length during diestrus. This pattern is flipped after repeated stress. In males, a lower dendritic length relative to females is increased after repeated stress. (**C**) Spine number of LAT neurons shifts across estrous, with higher number of spines during proestrus. This pattern is maintained after repeated stress, but with greater swings in spine number across estrous. In males, LAT neuron spine number is not greatly increased by repeated stress. Spine number of BA neurons shifts across estrous, with higher number of spines during diestrus. This pattern is flipped after repeated stress. In males, a lower number of BA neuron spines relative to females is greatly increased after repeated stress. (**D**) Expression of condition fear shifts across estrous, with higher freezing during diestrus. After repeated stress, conditioned freezing is uniformly high across estrous. In males, conditioned freezing is higher after repeated stress. Acquisition of fear extinction shifts across the estrous cycle, with better acquisition (lower freezing) in proestrus compared to diestrus. This pattern is flipped after repeated stress, with overall decreased acquisition of extinction. In males, acquisition of fear extinction is not greatly impacted after repeated stress.

The goals of these experiments were to test the effects of repeated stress on the activity of the BLA in females, and determine if stress disrupts the normal fluctuations of BLA activity and morphology that occur over the estrous cycle. In females, repeated stress reduced BLA neuronal firing rate, produced dendritic hypotrophy and a small shift in the distribution of spines on LAT neurons, while increasing conditioned freezing and slowing acquisition of extinction (Fig. 9). But in males, repeated stress increased BLA neuronal firing rate, produced BA dendritic hypertrophy and increased dendritic spines alongside increased conditioned freezing (Fig. 9). Despite different neuronal changes, both males and females displayed increased conditioned fear. It is possible that the sex differences in the neuronal changes observed might translate into subtle aspects of conditioned fear that were not examined here, as supported by differences in the effects of stress on extinction. We examined darting, but the occurrence of darts was rare, possibly due to differences in conditioning procedures or attributes of the conditioning chambers^67^. Alternatively, the neuronal changes might be more important for other BLA-related behaviors.

In female rats, BLA neurons exhibited different patterns of activity that were associated with specific days of the estrous cycle. Exposure to repeated stress interfered with these cycle stage-dependent patterns of BLA activity with the LAT and BA being differentially altered by stress (Fig. 9). Within the LAT, repeated stress disrupted the fluctuations of LAT neuron firing that occur over the estrous cycle. But stress enhanced the fluctuations of BA neuron firing over the estrous cycle and flipped the pattern of morphological changes in BA neurons that occurs over the estrous cycle. This disrupted pattern of neuronal activity and morphology paralleled the effects of repeated stress on BLA-dependent conditioned freezing and extinction. In control animals, there is greater conditioned freezing during diestrus compared to proestrus, but slower extinction in proestrus. Repeated stress dampened the changes in cued conditioned fear and flipped the changes of fear extinction that occur over estrous (Fig. 9). Because cued fear relies heavily on LAT and fear extinction involves the BA, these changes in the pattern of behaviors over estrous caused by repeated stress may be related to the parallel changes of LAT and BA neuronal function. Most studies of amygdala activation in humans after stress or depression find increased activity in males and females^68,69^. However, those studies usually lack the resolution to separate nuclei of the amygdala and seldom track ovarian phase. In fact, there are recent studies that suggest decreased amygdala activation can be observed in some female patients with depression^70,71^.

Biological rhythms are ubiquitous in mammals, and exert significant adaptive functions on feeding, drinking, sleeping and reproduction. The cascade of neural and humoral activity across the estrous cycle, particularly during proestrus/estrus, is important for ovulation and preparing the uterus for potential impregnation; as well as generating reproductive and social behaviors geared toward successful mating. Therefore the changes in amygdala activity that are observed here may have important ramifications for performance of social behaviors appropriate to different phases of the estrous cycle, specifically mating. The measured endocrine parameters, estradiol levels, uterine ballooning, and vaginal cell cytology, were not affected by stress; however, repeated stress did alter the pattern of BLA activity that normally occurs across the estrous cycle. Earlier studies^72,73^ demonstrated that disruption of BLA activity influences the expression of mating-related behaviors but has only modulatory effects on the timing of the luteinizing hormone (LH) with no significant impact on ovulation. Our data are in alignment with this in that stress-induced alterations in BLA activity were not associated with acyclicity as assessed by our measured parameters. The outcome is that, relative to coordinated changes in BLA function associated with a particular estrous phase in controls, BLA activity and function now align to different estrous phase, and presumably no longer acts to facilitate adaptive behaviors that would be congruent with the hormonal rhythm. It is not clear what drives the normal pattern of BLA function across the estrous cycle, though it may involve alterations in the sensitivity of the amygdala to sex hormones as has been previously demonstrated^47^. Furthermore, mechanisms contributing to the development of the abnormal patterning of LAT and BA neuronal function across estrous after stress are not known. These changes are not likely due to disruption of the estrous cycle *per se*, since estrous cyclicity continued unabated. One interesting hormonal response to stress was the overall increased circulating levels of estradiol on both days of the cycle without affecting the typical estrous pattern (higher on proestrus); stress-induced increases in estradiol have been noted in other studies^74^ (but see^75,76^). These elevated levels of estradiol could impact the BLA and therefore interfere with the normal cycle-related patterns of BLA activity and BLA function. Additionally, other sex hormones (such as progesterone) could also contribute to the altered firing patterns of LA and BA neurons at the different cycle times.

A tight association of gonadal hormone levels and limbic function has been observed across the estrous cycle and a mismatch between these rhythms is associated with the development of psychiatric disorders. Healthy women in the luteal phase display higher left amygdala reactivity than in their follicular phase, but this pattern of amygdala activity is reversed in women with premenstrual dysphoric disorder^77^, and the suppressive effects of estradiol on amygdala activation across the menstrual cycle is impaired in women with major depression^78^. While the functional importance of this mismatch in humans is not known, our results suggest that repeated stress can cause a similar mismatch.

Among its initial effects, stress triggers the hypothalamic-pituitary-adrenal (HPA) axis. Numerous studies demonstrate sex differences in the HPA axis and its response to stress^79–83^. Both human and rodent females have higher levels of glucocorticoids compared to males under normal physiological conditions^84–86^. In response to an acute stressor, females show a heightened glucocorticoid response, higher levels of ACTH, faster release of glucocorticoids and a prolonged duration of elevated glucocorticoid levels compared to males^86–89^. This could be the initial step in producing a sex difference in the effects of stress on BLA neurons.

Previous work has found that stress increases BLA neuron activity, excitability and spine number in males^38,39,42,61–63,90–93^. Several mechanisms have been uncovered to explain the effects of stress on BLA activity in males including increased excitatory input, decreased GABAergic regulation, and increased intrinsic membrane excitability^61,63,90–93^. One consequence of many of the proposed mechanisms in males is a stress-induced imbalance of neuronal inhibition and excitation. The balance between excitatory and inhibitory inputs determines BLA neuron firing. While this balance was not directly measured in the current study, a change in spine number implicates a change of excitatory synapses, while CV reflects the excitatory-inhibitory balance that promotes firing of BLA neurons. These measures fluctuate across the estrous cycle and repeated stress altered the pattern of fluctuation across the estrous cycle. This is preliminary support that a shift in the excitatory-inhibitory balance in females may contribute to the effects of stress on the BLA neurons.

Changes in BLA neuron spine density were examined to reveal if stress had an effect on spines at specific locations from the cell soma. Differences in spine density from proximal to distal locations from the cell soma may reflect impact on specific sources of afferents and will have different effects on the kinetic properties of excitatory input and synaptic integration^94–96^. Spine density was increased on BA neurons at distances closer to the soma in males^38,62^. In females, stress shifted spines toward more proximal locations and dampened the usual changes of LAT neurons spine number that occur over the estrous cycle. In BA neurons however, stress led to a reversal in the pattern of changes across estrous, such that spine number was greatest during proestrus (Fig. 9). Although the specific topography of excitatory inputs to dendrites in BLA neurons is still not clear, the differential pattern of the effects of stress on spine density in females and males may produce a sex difference in a specific source of, or response to, afferent input to BLA.

Repeat exposure to stress is often a precipitating factor in the onset of affective disorders. Females are two times more likely to suffer from affective disorders suggesting that there are sex-specific responses to stress in brain regions implicated in these disorders, such as the amygdala. Although the impact of stress on the characteristics and neuronal properties of amygdala neurons in males has been previously investigated (see above), this is the first study to demonstrate that stress has opposing effects on BLA neuron activity between females and males. These results may help explain the disparity in cued and contextual fear conditioning and extinction observed between sexes in response to stress and provide insight into a basis for sex differences in the effects of stress on emotion, and perhaps in affective disorders.

The datasets generated during and/or analyzed during the current study are available from the corresponding author on reasonable request.
